# Comparison of lipemia interference created with native lipemic material and intravenous lipid emulsion in emergency laboratory tests

**DOI:** 10.11613/BM.2024.020701

**Published:** 2024-04-15

**Authors:** Emel Çolak Samsum, Hatice Sürer, Serkan Bolat, Mehmet Şeneş, Doğan Yücel

**Affiliations:** 1 Medical Biochemistry, Ministry of Health Pursaklar State Hospital, Ankara, Turkey; 2 Medical Biochemistry, University of Health Sciences Ankara Training and Research Hospital, Ankara, Turkey; 3 Department of Medical Biochemistry, Sivas Cumhuriyet University, Sivas, Turkey; 4 Department of Medical Biochemistry, Lokman Hekim University, Ankara, Turkey

**Keywords:** lipemia, intravenous lipid emulsions, interference, clinical chemistry tests, preanalytical phase

## Abstract

**Introduction:**

This study aimed to investigate the effects of lipemia on clinical chemistry and coagulation parameters in native ultralipemic (NULM) and intravenous lipid emulsion (IVLE) spiked samples.

**Materials and methods:**

The evaluation of biochemistry (photometric, ion-selective electrode, immunoturbidimetric method), cardiac (electrochemiluminescence immunoassay method) and coagulation (the viscosity-based mechanical method for prothrombin time (PT), activated partial thromboplastin time (APTT), fibrinogen and the immunoturbidimetric method for D-dimer) parameters were conducted. In addition to the main pools, five pools were prepared for both types of lipemia, each with triglyceride (TG) concentrations of approximately 2.8, 5.7, 11.3, 17.0 and 22.6 mmol/L. All parameters’ mean differences (MD%) were presented as interferographs and compared with the desirable specification for the inaccuracy (bias%). Data were also evaluated by repeated measures of ANOVA.

**Results:**

Prothrombin time and APTT showed no clinically relevant interference in IVLE-added pools but were negatively affected in NULM pools

(P < 0.001 in both parameters). For biochemistry, the most striking difference was seen for CRP; it is up to 134 MD% value with NULM (P < 0.001) at the highest TG concentration, whereas it was up to - 2.49 MD% value with IVLE (P = 0.009). Albumin was affected negatively upward of 5.7 mmol/L TG with IVLE, while there was no effect for NULM. Creatinine displayed significant positive interferences with NULM starting at the lowest TG concentration (P = 0.028). There was no clinically relevant interference in cardiac markers for both lipemia types.

**Conclusions:**

Significant differences were scrutinized in interference patterns of lipemia types, emphasizing the need for careful consideration of lipemia interferences in clinical laboratories. It is crucial to note that lipid emulsions inadequately replicate lipemic samples.

## Graphical abstract

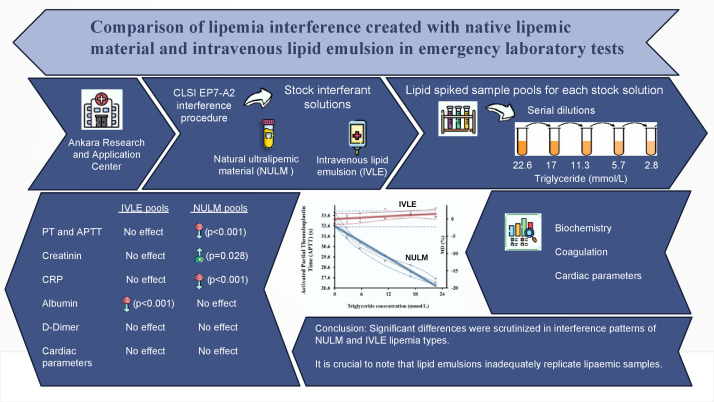

## Highlights

• After hemolysis and icterus, lipemia is one of the most common errors encountered in the preanalytical phase

• Mimicking lipemia is more challenging than other interference types because of the heterogenous property of lipids

• Intravenous lipid emulsions are not sufficient for mimicking lipemia

• Since almost all companies use intravenous lipid emulsion in lipemia interference studies, the results of these studies must be interpreted carefully by the laboratory experts

## Introduction

Interference is an effect that causes a clinically significant error in the measured analyte concentration due to the nature of the sample or another substance present in the sample (*1*). Lipemia is a rare but essential type of interference in the preanalytical phase (0.5-2.5%) (*2*). The most common causes of lipemia are impaired fasting, diet, alcohol intake, lipid metabolism disorders, total parenteral nutrition, some drugs and chronic diseases, such as diabetes mellitus, chronic renal failure and hypothyroidism (*3*). Lipemia-induced interference mechanisms are light scattering, light absorbance, electrolyte exclusion, partitioning of the analytes between polar and non-polar phases, physicochemical and biological effects (*2*). Lipoprotein particles can cause turbidity leading to light scattering. Consequently, lipemia can lead to substantial interference in photometers, specifically in turbidimetric and nephelometric methods based on light scattering (*4*). Light scattering can occur in all directions and its intensity depends on the number and size of lipoprotein particles and the measurement wavelength. Chylomicrons and very low-density lipoproteins (VLDL) are the primary factors responsible for turbidity (*5*, *6*). Moreover, lipoprotein particles can affect all results by leading to light absorbance in the 300-700 nm, where photometric measurements are conducted. However, absorbance is inversely proportional to the wavelength. As a result, lipemia tends to have a more substantial effect on methods that use lower wavelengths (*2*).

In interference studies, bilirubin and hemoglobin are added to the samples when evaluating icterus and hemolysis. Currently, we have no standard material to mimic native lipemia due to the heterogeneity of lipoproteins (*7*, *8*). Glick and colleagues used Intralipid (Fresenius Kabi, Bad Hamburg, Germany), one of the most commonly used intravenous lipid emulsion (IVLE) solutions, to create lipemic samples (*9*). However, IVLE solutions contain different components from native lipemic serum/plasma, such as soybean oil, egg yolk phospholipids and glycerin (*10*). One notable limitation of Intralipid is its particle size, with an average of 345 nm and a range from 200 to 600 nm. Therefore, it does not comprise large chylomicrons (up to 1000 nm) and large VLDL particles (35-200 nm). Moreover, the particles’ refractive index in Intralipid differs from lipoproteins (*8*). It is crucial to acknowledge that the composition and characteristics of synthetic lipid emulsions can vary and not all of them share the same limitations as Intralipid. Previous studies have shown conflicting results between the use of intralipid emulsions and natural lipemia (*11*-*13*). Most of the interference studies have been done with IVLE and lipemia interference in commercial products is also based on IVLE-spiked studies. Studies with native lipemic samples are limited (*12*, *14*-*17*).

This study aimed to investigate the effects of lipemia on clinical chemistry and coagulation parameters in native lipemic and IVLE spiked samples. Based on former studies, we hypothesized that natural lipemia would affect these parameters differently. Therefore, we planned to prepare an ultralipemic material from lipemic patient sera to mimic native lipemia (*18*).

## Materials and methods

This study was conducted in the Emergency Laboratory of the Biochemistry Department at Ankara Research and Application Center in accordance with the EP7-A2 and C56-A protocols by the Clinical and Laboratory Standards Institute (CLSI) (*1*, *19*). The study protocol was prepared following the Helsinki Declaration and approved by the Health Sciences University Ankara Training and Research Hospital Clinical Research Ethics Committee (Decision No:295/2020;10.07.2020).

### Preparation of stock interferents solutions

Two interferents, commercial lipid emulsion (Oliclinomel N-7, 1000 E (20%, Baxter Inc. Lessines, Belgium)) and the native ultralipemic material (NULM) prepared in-house, were used. To prepare NULM, a 100 mL serum pool was collected from approximately 40 residual lipemic serum samples (BD Vacutainer SST II Plus, 5 mL, Becton Dickinson, Franklin Lake, USA) with a triglyceride (TG) concentration > 22.6 mmol/L. The samples from patients who used IVLE or came from the intensive care unit were not included in the study to prevent IVLE contamination. Samples were not frozen, as freeze-thaw cycles would cause errors. They were stored at 2-8 °C for seven days by the stability of the lipids (*20*). Then, this pool was centrifuged three times at 45,000xg for 30 minutes in a Hanil Supra 21K (Hanil Scientific Inc., Gimpo, South Korea) refrigerated high-speed centrifuge. After each centrifugation, the supernatant lipid layer was collected carefully. Subsequently, only this lipid-rich portion underwent centrifugation in the following step. At the end of the third collection, we had 5mL of NULM.

### Preparation of sample pools

Two 50 mL serum pools were prepared by collecting 40 fresh and non-turbid residual serums (Vacutainer SST II Plus, 5 mL, Becton Dickinson, Franklin Lake, USA) with TG concentration < 0.99 mmol/L; one pool was designated for biochemical analytes, while the other was intended for cardiac analytes. All analyzed tests were within the reference range. Likewise, 20 residual plasma (4.5 mL, Becton Dickinson, Franklin Lake, USA), with coagulation analytes within reference ranges and no visible turbidity, were collected. A 50 mL plasma pool was prepared. The pools were stored at 2-8 °C. Biochemical and cardiac analytes were measured within seven days and coagulation analytes were measured within four hours.

### Addition of native ultralipemic material and intravenous lipid emulsion to pools

**Figure 1:**
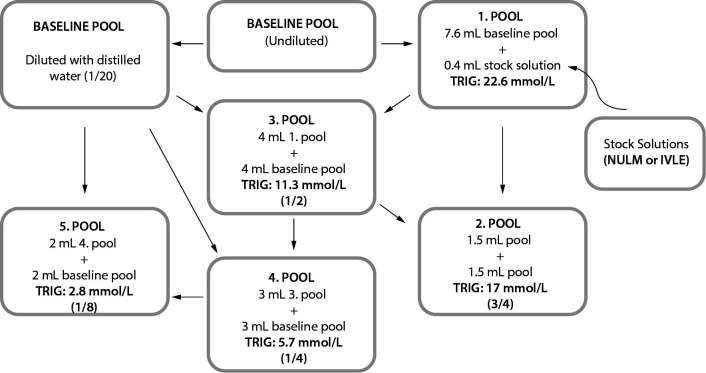
Flow chart of preparation of pools with different triglyceride (TG) concentrations. NULM - native ultralipemic material. IVLE - intravenous lipid emulsion.

According to the CLSI guideline C56-A, the highest TG concentration in the pools was determined to be 22.6 mmol/L by evaluating the high TG concentrations observed in lipemic samples in our laboratory. The stock solutions (NULM and IVLE) were concentrated at least 20 times the target concentration and when spiking, dilutions did not exceed 5% to minimize the deterioration of the sample matrices. For the first pools, 7.6 mL baseline pools spiked with 0.4 mL stock solutions. The remaining baseline pools were diluted with distilled water at the same ratio (1/20) to compensate for pools dilutions. Five pools were prepared for both NULM and IVLE types of lipemia by taking samples in the amounts indicated in Figure 1 from the first and baseline pool tubes. Each pool was designed to have a different TG concentration, approximately 2.8, 5.7, 11.3, 17.0 and 22.6 mmol/L (Figure 1).

### Investigated analytes

In the serum pools N-terminal pro brain natriuretic peptide (NT-proBNP), creatine kinase MB isoenzyme mass (CK-MB) and high-sensitivity troponin T (hs-TnT) were investigated by the sandwich-type electrochemiluminescence immunoassay (ECLIA) method on the Roche Cobas e411 (Roche, Basel, Switzerland) analyzer.

Also, biochemical analytes measured by Roche Cobas 6000 c501 (Roche, Basel, Switzerland) and ABL800-FLEX blood gas analyzer (Radiometer Medical ApS, Copenhagen, Denmark) are given in Table 1.

**Table 1:** Investigated biochemistry parameters and methodology

Analytes	Methodology	Wavelength (sub/main, nm)
	**Roche Cobas 6000 c501**	
Alb	Bromocresol green (BCG)	505/570
ALT	IFCC, UV, without P5P	700/**340**
AST	IFCC, UV, without P5P	700/**340**
AMY	IFCC, 4,6-ethylidene-(G7) p-nitrophenyl-(G1) α-D-maltoheptaoside (ethylidene-G7PNP)	700/415
CREA	Compensated Jaffe method	570/505
DBIL	Diazo method	800/546
TBIL	Diazo method	600/546
Ca	5-nitro-5’- methyl-BAPTA (NM-BAPTA) method	376/**340**
CHE	Butyrylthiocholine	700/415
LD	IFCC, UV, Lactate-pyruvate conversion	700/**340**
GGT	IFCC, L-γ-glutamyl-3-carboxy-4-nitroanilide (GGCN)	700/415
Glc	Hexokinase	700/**340**
CK	IFCC, UV, NAC activated	546/**340**
Mg	Xylidyl blue	505/600
Phos	UV, Ammonium molybdate	700/**340**
TP	Biuret method	700/546
UA	Enzymatic, uricase	700/546
Urea	UV, Urease/Glutamate dehydrogenase (GLDH)	700/**340**
CRP	Immunoturbidimetric	800/570
Na, K, Cl	Indirect ISE	/
	**Radiometer ABL800 FLEX**	
Na, K, Ca^2+^	Direct ISE	/
	**Roche Cobas 8000 c702**	
TG	Enzymatic method (using glycerol blank)	700/500
CHOL	Cholesterol esterase, oxidase, peroxidase	700/505
HDL	Homogeneous colorimetric method	700/600
LDL	Homogeneous colorimetric method	700/600
Serum index	Absorbance measurements at bichromatic wavelength pairs	For lipemia 700/660
Values in bold and underlined indicate wavelengths at which lipids show high absorbance. IFCC - International Federation of Clinical Chemistry and Laboratory Medicine. UV - ultraviolet. P-5-P - pyridoxal-5-phosphate. NAC - N-acetyl cysteine. ISE - ion selective electrode. Alb - albumin. ALT - alanine aminotransferase. AST - aspartate aminotransferase. AMY - amylase. CREA - creatinine. DBIL - direct bilirubin. TBIL - total bilirubin. Ca - calcium. CHE - cholinesterase. LD - lactate dehydrogenase. GGT - gamma-glutamyltransferase. Glc - glucose. CK - creatine kinase. Mg - magnesium. Phos - inorganic phosphate. TP - total protein. UA - uric acid. CRP - C-reactive protein. Na - sodium. K - potassium. Cl - chloride. Ca^2+^ - ionized calcium. TG - triglyceride. CHOL - total cholesterol. HDL - high-density lipoprotein cholesterol. LDL - low-density lipoprotein cholesterol.		

In the citrated plasma pool, prothrombin time (PT), activated partial thromboplastin time (APTT), fibrinogen (Fbg) and D-dimer (DD) were investigated on the Stago STAR Max (Diagnostica Stago SAS, Asnières Sur Seine, France) analyzer. D-dimer was also measured on Roche Cobas 6000 c501 analyzer (Roche, Basel, Switzerland). Fibrinogen, PT and APTT were measured using the viscosity-based mechanical method and DD was measured using the immunoturbidimetric method in both analyzers.

### Lipid and serum index measurements

Triglyceride, total cholesterol (CHOL), high-density lipoprotein cholesterol (HDL), low-density lipoprotein cholesterol (LDL) and serum index measurements were performed on the Roche Cobas 8000 c702 (Roche, Basel, Switzerland) analyzer to determine the degree of lipemia and turbidity in the pools (Table 1, Supplementary table 1). Furthermore, lipoprotein gel electrophoresis for NULM was conducted (Sebia Hydrays, Paris, France).

### Statistical analysis

IBM SPSS Statistics Windows version 22.0 (IBM Corp., Armonk, New York, USA) program was used for statistical analysis. The data distribution was determined by Shapiro-Wilk tests and by examining histogram graphs. Parametric data were given as mean ± SD. A repeated measure ANOVA test was used to determine whether there was a significant difference between the pools for all analytes. The statistical significance level was considered as P < 0.05. Microsoft Excel (Microsoft Corp., Redmond, USA) was used to prepare interferographs. In the study, the analytes were measured three times in each pool and the averages of these measurements were used in the calculations. For each analyte, the mean percentage difference (MD%) in lipid-added pools relative to the baseline pool was calculated, MD = [(C_1_ - C_0_) / C_0_] x 100; (C_1_, mean analyte concentration in the lipid-added pool; C_0_, mean analyte concentration in baseline pool). Interferographs were arranged the way interferent concentrations were on the x-axis, MD% values were on the right side of the y-axis and mean values of analytes were on the left side of the y-axis. Significant interference was considered when the MD exceeded the desirable specification for inaccuracy (± bias%) obtained from the biological variation data, which was available from a database formed by Ricos *et al*. (*21*). Desirable total allowable error (TEa) derived from intra- and inter-individual biological variation data might serve to assess the clinical relevance of interference, encompassing two crucial components: desirable bias and desirable imprecision. In interference studies involving multiple measurements for each pool, the impact of imprecision is minimized. Consequently, as in this study, desirable bias values function as a practical interference budget.

## Results

The concentrations of TG in stock interferent solutions were 469 and 501 mmol/L for NULM and IVLE, respectively. The lipoprotein electrophoresis for NULM revealed the following composition: 12% chylomicron, 63% LDL, 8.6% VLDL and 15% HDL.

**Figure 2:**
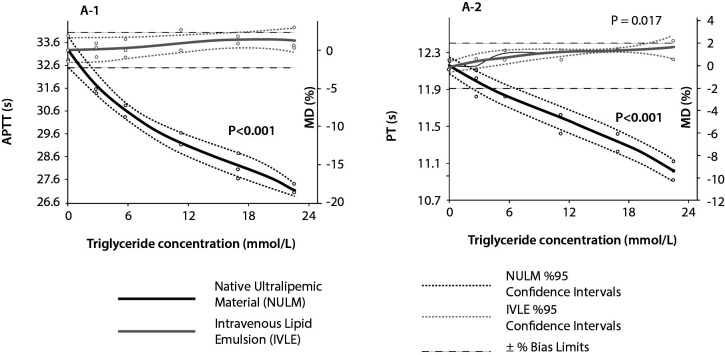
Lipemia interferographs of coagulation parameters. MD - mean difference. APTT - activated partial thromboplastin time. PT - prothrombin time.

**Figure 3:**
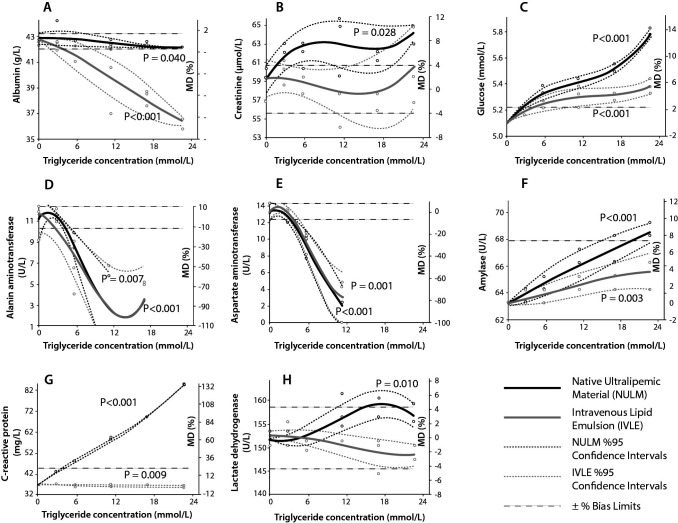
Lipemia interferographs of biochemistry parameters. MD - mean difference.

**Figure 4:**
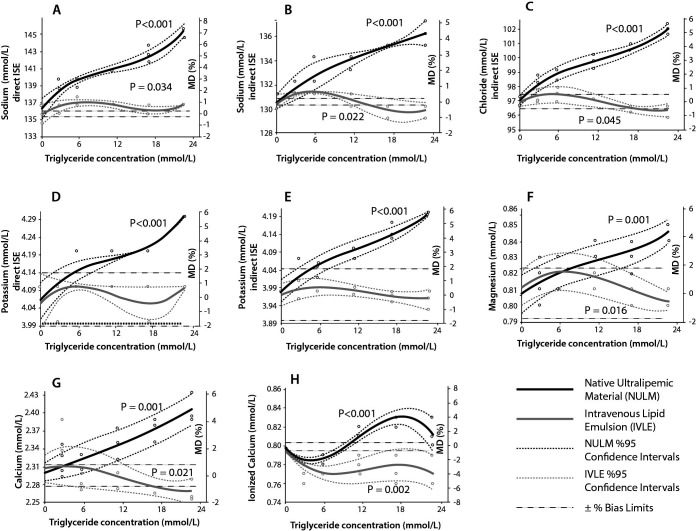
Lipemia interferographs of biochemistry parameters. MD - mean difference.

The measurements of all analytes in baseline pools and the corresponding calculated MD% values for all lipid concentration are outlined in Table 2. Lipemia interferographs are shown in Figures 2, 3 and 4.

**Table 2:** Mean percentage difference (MD%) between baseline pool and lipid spiked pools for different triglyceride concentrations and two lipemia types: native ultralipemic material (NULM) and intravenous lipid emulsion (IVLE)

**Analytes**	**Baseline pool** **(mean ± SD)**	**NULM-1** **(MD%)**	**NULM-2** **(MD%)**	**NULM-3** **(MD%)**	**NULM-4** **(MD%)**	**NULM-5** **(MD%)**	**P**	**IVLE-1** **(MD%)**	**IVLE-2** **(MD%)**	**IVLE-3** **(MD%)**	**IVLE-4** **(MD%)**	**IVLE-5** **(MD%)**	**P**	Bias (%)**
TG, mmol/L		2.8	5.7	11.3	17.0	22.6		2.8	5.7	11.3	17.0	22.6		
PT, sec	12.1 ± 0.1	- 1.4	- 2.5*	- 4.9	- 6.6	- 9.3	< 0.001	0.0	1.1	1.1	1.4	1.7	0.017	2
APTT, sec	33.3 ± 0.6	- 4.5*	- 8.2	- 11.8	- 15.3	-18.2	< 0.001	0.1	0.2	0.9	1.3	1.3	0.601	2.3
Fbg, g/L	3.2 ± 0.1	0.9	3.4	0.0	- 2.9	2.3	0.307	1.3	1.1	- 1.2	- 1.8	0.0	0.728	4.8
DD (Stago), mg/L	0.63 ± 0.08	- 2.7	8.5	16	14.4	- 17	0.270	- 5.3	- 5.9	6.4	7.5	- 4.3	0.793	8.82
DD (Roche), mg/L	0.64 ± 0.01	- 2.6	- 4.2	- 0.5	- 9.4	0.0	0.465	- 2.1	- 0.5	0.5	- 2.1	- 4.7	0.465	8.82
CK-MB, ng/mL	2.4 ±0.03	2.7	3.4	3	3.4	2.3	0.648	- 2.2	- 0.7	- 0.5	2.1	- 1.0	0.071	14.88
NT-proBNP, ng/L	834 ± 16.5	1.9	0.4	2.8	3.5	2.2	0.299	0.5	2.6	1.5	1.5	2.9	0.295	4.7
hs-TnT, ng/L	14 ± 0.2	- 7.1	- 10.7	- 2.3	- 4.1	0.7	0.123	- 4.6	- 5.8	- 4.2	- 9.7	- 1.9	0.006	23.7
Alb, g/L	42.6 ± 0.3	1.80	- 0.31	- 0.63	- 0.55	1.33	0.04	- 0.63	- 2.35*	- 7.75	- 10.33	- 14.95	< 0.001	1.43
ALT, U/L	11 ± 2	2.06	- 22 (A)	- 61 (A)	A	A	0.007	2.06	- 40 (A)	- 58 (A)	- 54 (A)	A	< 0.001	11.48
AST, U/L	13 ± 1	- 5.76	- 30 (A)	- 75 (A)	A	A	< 0.001	- 1	- 28 (A)	- 65 (A)	A	A	0.001	6.54
AMY, U/L	63 ± 0	1.06	1.59	5.29	5.82	8.47*	< 0.001	0.53	1.06	2.12	3.17	3.70	0.003	7.4
CREA, µmol/L	59 ± 2	6.53*	5.03	7.54	6.03	9.05	0.028	2.01	0.00	- 3.02	- 0.50	2.51	0.749	3.96
DBIL, µmol/L	2.2 ± 0.2	35*	75	128	184	333	< 0.001	59*	98	174	254	309	< 0.001	14.2
TBIL, µmol/L	5 ±1	0.97	- 16.3	2.47	4.19	- 9.77	0.809	10.3	- 9.56	- 20.2	- 17.3	8.92	0.183	8.95
Ca, mmol/L	2.29 ± 0.01	1.30*	0.76	2.21	3.37	4.78	0.001	1.92*	- 0.51	0.11	- 0.76	- 1.23	0.021	0.82
CHE, U/L	7252 ± 91	1.73	1.42	3.19	3.45	3.20	0.011	0.82	- 0.31	- 0.83	- 1.20	- 0.60	0.101	4.8
LD, U/L	152 ± 1.5	- 0.22	- 0.22	3.52	4.18	3.50	0.01	1.10	- 0.88	- 0.66	- 2.20	- 2.42	0.126	4.3
GGT, U/L	17± 1	6.00	2.00	6.00	2.00	0.00	0.141	8.00	4.00	6.00	6.00	4.00	0.491	11.06
Glc, mmol/L	5.1 ± 0	2.17	4.35*	6.16	8.00	13.41	< 0.001	1.81	2.54*	3.99	3.90	5.43	< 0.001	2.34
CK, U/L	79 ± 0	- 1.69	0.84	1.69	0.84	4.60	0.016	- 1.27	- 1.27	- 1.69	- 1.69	- 2.53	0.772	11.5
Mg, mmol/L	0.81 ± 0.01	0.00	1.23	2.88*	2.47	4.53	0.001	1.65	0.41	1.65	- 0.41	- 0.82	0.016	1.8
Phos, mmol/L	1.12 ± 0.03	- 0.57	- 1.34	- 1.44	- 2.49	- 2.30	0.570	- 1.15	- 2.78	- 4.02*	- 6.60	- 1.24	0.005	3.38
UA, mmol/L	0.24 ± 0.01	- 1.63	0.81	0.00	1.63	1.63	0.620	- 4.07	- 2.44	- 0.81	- 5.69*	- 5.69	0.272	4.87
Urea, mmol/L	4.3 ± 0.1	- 0.13	2.49	4.32	2.75	3.80	0.004	1.31	- 0.52	- 0.52	- 1.18	- 3.01	0.150	5.57
CRP, mg/L	36.1 ± 0.2	16.2	30.4*	60.7	89.8	133.6	< 0.001	0.83	- 1.48	- 1.39	- 1.57	- 2.49	0.009	21.8
Na (I-ISE), mmol/L	130 ± 0.6	0.77*	1.79	2.56	3.58	4.35	< 0.001	0.51*	0.51	0.26	- 0.51	- 0.51	0.022	0.23
K (I-ISE), mmol/L	4.0 ± 0.03	1.76	1.85*	3.02	4.45	5.71	< 0.001	0.34	0.59	0.25	- 0.08	- 0.34	0.490	1.81
Cl (I-ISE), mmol/L	97 ± 0.3	1.55*	1.79	2.93	3.86	5.24	< 0.001	0.31	0.55*	0.1	- 0.52	- 0.62	0.045	0.5
Na (D-ISE), mmol/L	136 ± 1	2.70*	2.45	3.68	5.15	7.10	< 0.001	0.49*	0.98	0.74	0.25	0.74	0.034	0.23
K (D-ISE), mmol/L	4.1 ± 0.6	0.82	1.64	3.28*	3.28	5.74	< 0.001	0.00	0.82	0.82	- 0.82	0.82	0.197	1.81
iCa^2+^ (D-ISE), mmol/L	0.8 ± 0	- 2.92*	- 1.25	1.67	3.33	1.67	< 0.001	- 4.17*	- 2.92	- 2.5	- 3.33	- 3.33	0.002	0.6
*Mean difference (MD%) exceeds bias% decision limit. **Desirable specification for the inaccuracy (bias%) data derived from biological variation for all analytes. A - instrument did not report AST and ALT because the interference was too high. (A) - instrument reported a result with a ﬂag. TG - triglycerides. PT - prothrombin time. APTT - activated partial thromboplastin time. Fbg - fibrinogen. DD - D-dimer. CK-MB - creatine kinase MB isoenzyme mass. NT-proBNP - N-terminal pro brain natriuretic peptide. hs-TnT, high - sensitivity troponin T. Alb - albumin. ALT - alanine aminotransferase. AST - aspartate aminotransferase. AMY - amylase. CREA - creatinine. DBIL - direct bilirubin. TBIL - total bilirubin. Ca - calcium. CHE - cholinesterase. LD - lactate dehydrogenase. GGT - gamma-glutamyl transferase. Glc - glucose. CK - creatine kinase. Mg - magnesium. Phos - inorganic phosphate. UA - uric acid. CRP - C - reactive protein. Na - sodium. K- potassium. Cl - chloride. I-ISE - indirect ion-selective electrode. D-ISE - direct ion-selective electrode. iCa2+ - ionized calcium.														

Significant negative interferences were detected for PT and APTT in NULM spiked pools which were proportional to TG concentrations (in both P < 0.001). In contrast, significant positive interference for PT was determined in IVLE spiked pools (P = 0.017), but the interference level did not exceed the bias% limit up to TG concentrations of 22.6 mmol/L.

Despite statistically significant negative interference for hs-TnT in IVLE spiked pools (P = 0.006), interference did not exceed the bias% decision limit.

Significant positive interferences for amylase (AMY) were determined in both lipemia types, but the interference exceeded the bias% limit for only NULM pools at > 17 mmol/L TG. Although cholinesterase (CHE), creatin kinase (CK) and lactate dehydrogenase (LD) were positively affected in NULM spiked pools (P = 0.011, 0.016, 0.010, respectively), only LD exceeded the bias% limit.

Significant positive interferences were determined for calcium (Ca) and magnesium (Mg) in NULM spiked pools, whereas significant negative interferences were obtained in IVLE spiked pools.

Significant negative interferences for albumin (Alb) were determined in both lipemia types, but the interference in NULM spiked pools was not clinically significant. The negative interference for Alb has exceeded the bias% limit at TG > 5.7 mmol/L concentration.

There was positive interference for CREA in NULM spiked pools (P = 0.028), which exceeded the %bias limit at > 2.8 mmol/L concentration. There was no interference for CREA in IVLE spiked pools.

We found that lipemia had statistically significant positive interference on C-reactive protein (CRP) in NULM pools, while a negative interference was in IVLE pools. However, interference was not clinically meaningful in IVLE pools. The interference in NULM pools has exceeded the bias% limit at > 5.8 mmol/L TG concentration.

Significant positive interferences were obtained for direct ion-selective electrode (ISE) (sodium (Na) and potassium (K)) and indirect-ISE measurements (Na, K and chloride (Cl)) in NULM pools (P < 0.001 in all). There was biphasic interference for ionized Ca (iCa^2+^) in NULM pools. No interference was determined for direct and indirect K in IVLE pools. Direct Na measurement was affected positively, while iCa^2+^ was affected negatively in IVLE pools.

## Discussion

This study aimed to assess the differences in interferences induced by natural lipemia and those caused by IVLE in diverse analytes. Our investigation revealed variations in interference patterns between natural lipemia and IVLE across most parameters, aligning with our initial hypothesis and showcasing differences in either direction, magnitude or both.

Lipemia causes interferences in coagulation parameters with different mechanisms such as analytical effects, primarily seen in optical methods, biological effects and direct alteration of primary and secondary hemostasis components (*22*). The researchers using IVLE show that lipemia does not affect the mechanical measurement procedure we used in our study (*23*, *24*). The fact that we found significant negative interferences with NULM in contrast to IVLE in our study confirms that lipid emulsions are insufficient to detect the biological interference that lipemia will cause for PT and APTT. In our research, negative interferences might stem from two reasons. The first one is that long-chain saturated fatty acids at the interface of lipoprotein residues can activate the contact system of *in vitro* coagulation (factor XII-intrinsic pathway) by providing a contact surface. Active products from this pathway (factor XIIa, factor IXa) can trigger factor VII, a significant component of the extrinsic pathway (*25*, *26*). The second one is that the movement of the magnetic bead might be mechanically restricted due to the increased viscosity.

In our study, DD was measured in two different analyzers using the immunoturbidimetric method. Since lipemia primarily causes interference through light absorbance and scattering, turbidimetric and nephelometric methods are expected to be the most affected (*2*, *4*, *27*). However, in reviewing the literature, there are studies in which DD measurement is unaffected (*28*, *29*). Although measured by immunoturbidimetric method, we did not observe any significant interference, similar to these studies. Studies with natural lipemic materials at different DD concentrations are needed to clarify this issue.

C-reactive protein is another immunoturbidimetric test. Similar to the effect of DD, it is stated in the literature and the kit inserts that there was no interference up to 1000 lipemia index for CRP measurement (*7*). However, our study observed significant positive interference with NULM commencing at the lowest TG concentration and proportional to TG concentration. The fact that one of the immunometric measurements is affected while the other is not may be because the analyses are performed at different wavelengths. On the Roche analyzer, DD is measured at 800 nm and CRP is measured at 570 nm. C-reactive protein, which is measured at lower wavelength, is affected by lipemia.

Cardiac parameters were determined by ECLIA, based on sandwich immunoassay, which contains two antibodies and has detailed washing steps (*30*). Due to these features, its sensitivity is high, and it has been observed that there is no lipemia interference as in previous studies (*31*, *32*). It was also consistent with the manufacturer’s declaration, which says there is no interference up to the 1500 lipemia index value (Supplementary table 2).

Interference evaluations were made for biochemistry parameters using IVLE on the Roche Cobas 6000 analyzer and these data were published before (*31*, *32*). Our results were evaluated by comparison with these studies and, where available, with test-based studies.

Statistically and clinically significant negative interference was detected for alanine aminotransferase (ALT) and aspartate aminotransferase (AST), consistent with previous studies (*14*, *31*, *32*). When lipemia index values were 159 and 174 for NULM and IVLE, respectively, the negative interference amount exceeded the 10% limit, conforming to the manufacturer’s declaration (Supplementary table 2). As in the Zeng *et al.* study, IVLE was observed to cause a sharper decrease in ALT than NULM (2% to - 40%, 2% to - 22%, respectively) (*14*). In future studies, preparing an additional pool of 2.8 and 5.7 mmol/L TG will help determine the interference initiation point in more detail. As the turbidity increased, no results were obtained because no signal could be received from the device. This started at lower TG concentrations with NULM than IVLE. Interference data are given in kit inserts according to IVLE. This means that in practice results cannot be obtained from more patients because of not receiving signals.

Studies on positive or negative interferences of AMY have been reported in the literature (using IVLE) (*33*, *34*). It has been reported in the kit package insert that it will be negatively affected after the 1500 lipemia index (10%). In our study, up to approximately 600 lipemia index values were examined in the highest pool. Contrary to the manufacturer’s declaration, we observed positive interference with both types of lipemia. Specifically, the NULM pool showed positive interference at an index of 600, surpassing the bias%, although not exceeding the 10% limit (Supplementary table 2).

The interference of lipemia is expected to increase as the wavelength decreases and the tests performed in the ultraviolet (UV) region are more affected (*8*). While this effect was observed for ALT and AST measured in the UV region, this distinction was not fully observed for some other tests. While gamma-glutamyltransferase (GGT) and CHE measured in the visible region were not affected by lipemia as expected, contrary to expectations, CK and LD measured in the UV region were unaffected by lipemia. These differences may be because the ratio of the sample volume used in the reaction to the total reaction volume differs between biochemistry analytes. No interference exceeding the 10% limit was observed for CK, LD, GGT and CHE in the IVLE pools, confirming the kit data up to 600 lipemia index. Nevertheless, LD displayed statistically significant positive interference in NULM pools, contrary to the manufacturer’s declaration (Supplementary table 2).

Negative or positive interferences have been shown in the literature for Ca, Mg and inorganic phosphate depending on the device, method and reagents (*3*, *33*-*35*). Although significant negative interference was observed in all three, we did not observe any interference exceeding the 10% limit with IVLE, which is consistent with the kit insert. Phosphorus was not significantly affected by NULM, Ca measured in the UV region was significantly positively affected as expected, while Mg measured in the visible region was positively affected, similar to other studies in the literature, contrary to expectations (*31*, *32*, *34*).

Studies show that Alb is negatively or positively affected or not. The kit insert observed significant negative interference with IVLE (*31*, *32*, *34*, *35*). Our findings were compatible with the manufacturer declaration in the IVLE pool. Negative interference observed in our study outstripped the 10% limit at similar lipemia index values in the insert (478 and 550, respectively) (Supplementary table 2). In contrast, no clinically significant effect was observed with NULM, although statistically significant.

Previous studies using IVLE for CREA have reported generally negative interference. The kit data published by Roche Diagnostics states that after the lipemia index value of 800, variable interference can be observed in the positive or negative direction (*3*, *7*, *34*). Ali *et al*. observed this biphasic effect in CREA in their study in which they evaluated lipemia interference using IVLE in 24 biochemistry parameters on a Roche Cobas 6000 analyzer (*32*). In our study, maximum lipemia index values of 614 and 633 were reached in pools with NULM and IVLE added. No effect was observed in the IVLE pool, consistent with the kit insert. A positive effect was observed in the NULM pool starting from 2.8 mmol/L TG. It was thought that an increase in CREA was detected similar to that of Ali *et al.* but at lower concentrations with NULM.

In direct bilirubin (DBIL), interference was observed in both types of lipemia, consistent with those reported in previous studies (*7*, *31*). This interference in DBIL exceeded the 10% limit even at the lowest TG concentration (2.8 mmol/L, around a lipemia index of 90), far below the 750 reported in the kit insert. This effect in DBIL may be due to the minor differences between absorbance measurements.

Negative and positive interferences have been reported for glucose in several studies. In our study, positive interferences consistent with the kit insert were observed in both types of lipemia. However, interference was higher with NULM than IVLE and had an early onset, passing the 10% limit at 633 lipemia index value, which is lower than the index value of 1000 given in the manufacturer declaration (*32*, *34*, *35*).

In clinical laboratories, electrolytes are most often measured by the potentiometric measurement method based on ISE. Blood gas analyzers measure directly without dilution, while autoanalyzers use the indirect method of diluting samples before measurement. In this method, conditions such as lipid disorders and hypo- or hyperproteinemia may lead to incorrect readings of electrolyte results due to the “electrolyte exclusion effect”. In cases of hyperlipidemia and hyperproteinemia, Na, K and Cl are measured as falsely low by the indirect method, whereas the direct method is unaffected (*2*). As in the study of Chopra *et al*., Na and K values measured by the direct method were higher than the autoanalyzer results (*36*). Contrary to the information in the literature and manufacturer declaration, positive interference was observed with both methods in pools to which NULM was added. Although TG concentrations are close, CHOL, HDL and LDL concentrations of NULM are approximately ten times higher than those of IVLE (Supplementary table 3). In this case, it was thought there must be another effect from exclusion that the electrode response may have changed due to the contamination of the ion-bound membrane surface with these compounds in both methods (*37*). In future studies, pools prepared with NULM should also be examined more thoroughly by measuring non-TG lipid concentrations.

This study has some limitations. Firstly, the amount of NULM we prepared was limited due to the small number of lipemic samples. Therefore, we could only evaluate the analytes at a single medical decision concentration. It will be more enlightening to evaluate different concentrations in future studies. Secondly, we could not evaluate how TG concentrations > 22.6 mmol/L will affect analytes due to the small number of samples and difficulties in preparing NULM.

In conclusion, our study compared interferences caused by natural lipemia and lipid emulsion across multiple analytes. It is crucial to note that IVLE does not accurately replicate lipemic patient samples. Significant differences were observed in interference patterns, reinforcing the need for careful consideration of lipemia interferences in clinical laboratories. These findings highlight the importance of tailored interference studies using natural lipemic samples to enhance result accuracy and ensure effective patient care.

## Data Availability

All data is given in the manuscript and supplemental materials.
